# Quality of Antimalarials at the Epicenter of Antimalarial Drug Resistance: Results from an Overt and Mystery Client Survey in Cambodia

**DOI:** 10.4269/ajtmh.14-0391

**Published:** 2015-06-03

**Authors:** Shunmay Yeung, Harriet L. S. Lawford, Patricia Tabernero, Chea Nguon, Albert van Wyk, Naiela Malik, Mikhael DeSousa, Ouk Rada, Mam Boravann, Prabha Dwivedi, Dana M. Hostetler, Isabel Swamidoss, Michael D. Green, Facundo M. Fernandez, Harparkash Kaur

**Affiliations:** Department of Global Health and Development, Faculty of Public Health and Policy, LSHTM, London, United Kingdom; Worldwide Antimalarial Resistance Network (WWARN), Centre for Tropical Medicine, University of Oxford, United Kingdom; National Center for Parasitology, Entomology and Malaria Control, Phnom Penh, Cambodia; Medecins sans Frontieres,Department of Global Health and Development, Faculty of Public Health and Policy, LSHTM, London, United Kingdom; Clinical Research Department, Faculty of Infectious and Tropical Disease, LSHTM, London, United Kingdom; Georgia Institute of Technology, School of Chemistry and Biochemistry, Atlanta, Georgia; Clinical Research Department, Faculty of Infectious and Tropical Disease, LSHTM, London, United Kingdom; Division of Parasitic Diseases, Centers for Disease Control and Prevention, Atlanta, Georgia

## Abstract

Widespread availability of monotherapies and falsified antimalarials is thought to have contributed to the historical development of multidrug-resistant malaria in Cambodia. This study aimed to document the quality of artemisinin-containing antimalarials (ACAs) and to compare two methods of collecting antimalarials from drug outlets: through open surveyors and mystery clients (MCs). Few oral artemisinin-based monotherapies and no suspected falsified medicines were found. All 291 samples contained the stated active pharmaceutical ingredient (API) of which 69% were considered good quality by chemical analysis. Overall, medicine quality did not differ by collection method, although open surveyors were less likely to obtain oral artemisinin-based monotherapies than MCs. The results are an encouraging indication of the positive impact of the country's efforts to tackle falsified antimalarials and artemisinin-based monotherapies. However, poor-quality medicines remain an ongoing challenge that demands sustained political will and investment of human and financial resources.

## Introduction

Historically, the Thai–Cambodia border has been a focal point for the development of antimalarial resistance.^1^ In response to the development of multi-drug resistant malaria, in 2000, Cambodia became the first countries to make artemisinin-based combination therapy (ACT), the national first-line treatment of uncomplicated *Plasmodium falciparum* (*Pf*) malaria. Since co-formulated ACTs were not commercially available at the time, artesunate and mefloquine were imported and packaged as age–weight-specific co-blister packs by the Cambodian Ministry of Health with support from the World Health Organization (WHO). These medicines were provided for free through public health facilities as “A + M” and socially marketed at a subsidized price through private sector under the brand name Malarine in recognition of the important role the private sector plays as a source of antimalarial treatment.^2^–^6^

In 2008, evidence of artemisinin-resistant *Pf* was reported on the Thai–Cambodia border.^7^,^8^ The repeated emergence of antimalarial drug resistance in this area is likely to be due to a number of contributing factors including the genetic epidemiology of the parasite itself.^9^ However, drug pressure exerted on the parasite population over time is likely to be a key driver. Drug pressure selects for relatively resistant parasites particularly when parasites are exposed to an antimalarial on its own as a monotherapy and to subtherapeutic concentrations. Subtherapeutic concentration of drugs can be due to different factors. The dose prescribed or sold by the provider may be inadequate; the right dose might be prescribed but poorly adhered to by the patient; there may be poor bioavailability; or the medicine may be of poor quality, containing inadequate doses of the active pharmaceutical ingredient (API).

As well as contributing to the development of drug resistance, poor quality medicines also have a direct effect on the patients who take them and can lead to treatment failure, severe disease and death as well as increased economic burden. Medicines may be of poor quality at time of purchase for a number of reasons. They may be falsified medicines (also known as spurious/falsely labeled/falsified/counterfeit [SFFC]) that are deliberately and fraudulently mislabeled, with respect to identity and/or source; they may be substandard medicines that are produced by manufacturers authorized by regulatory authorities, but which do not meet quality specifications standards set for them^10^; or they may be medicines manufactured according to quality specifications but which have degraded during transport and storage.

Falsified medicines have achieved most attention and the presence has been widely reported globally^11^–^15^ and in tropical countries, antimalarials have been particularly targeted by criminals.^16^,^17^ Studies in southeast Asia previously reported widespread availability of falsified “artesunate” containing no active ingredient and with up to 16 versions of the falsified holograms on the packaging.^4^,^18^ However, most of previous studies have not used random sampling and therefore in most cases the actual prevalence of falsified and poor-quality antimalarials is unknown.^19^

In Cambodia, falsified medicines as well as artemisinin-based monotherapies have previously been widely reported.^20^–^24^ Since then, there have been many initiatives focused on cracking down on poor-quality medicines including as a key component of the recent Artemisinin Resistance Containment program.^25^ To reduce drug pressure, a ban on oral artemisinin-based monotherapies was implemented, as strengthening of the drug regulatory and enforcement capacity.^25^–^27^ “containment zones” were defined, according to the level of drug resistance suspected and the first-line treatment of *Pf* malaria was switched from co-blistered artesunate and mefloquine to co-formulated dihydroartemisinin–piperaquine starting with Zone 1 in 2010, and later nationwide.

Recent surveys in Cambodia suggest that there has been a significant decrease in the availability of oral artemisinin-based monotherapies in the private sector.^6^ However, there are little recent data on the prevalence of poor-quality antimalarials. The primary aim of this study was therefore to provide robust estimates of the quality of artemisinin-containing antimalarials (ACAs) available in Cambodia and an examination of the risk factors associated with poor quality. In addition, despite guidelines,^28^ it is not clear what is the most suitable approach for procuring medicine samples for the analysis of drug quality. The secondary objectives of this study included a comparison of alternative approaches to procuring drugs by comparing the type and quality of malaria treatments bought through open interviews of private providers with those purchased covertly by mystery clients (MCs).

## Methods

### Study design.

This study was carried out in malaria-endemic areas of Cambodia as part of a study which used a mixed methods approach to studying how antimalarial drugs and malaria rapid diagnostic tests (mRDTs) are used in the private sector. The study included a census survey of private providers, MC study, observational study of the use of mRDTs and the quality of mRDTs transported and stored under field conditions.^29^ In this paper, we report the findings of the laboratory analysis of ACAs collected during the census survey and MC study. We define ACAs as any drug containing an artemisinin derivative (i.e., artesunate, artemether, artemisinin, or dihydroartemisinin) either as a monotherapy or in combination with a partner drug.

Results from the analysis of mRDT quality will be presented separately.

### Site selection.

The primary sampling unit for the selection of outlets was the “Health Centre Catchment Area”— the town and villages within the catchment areas of the health center. Health centers in Cambodia with more than 100 malaria cases in the previous year were stratified into those within containment areas (i.e., Zone 1 or 2, *N* = 55) or the noncontainment area (i.e., Zone 3, *N* = 29). From each strata, six health centers were randomly selected using a random number generator resulting in 12 health centers in total representing 10.9% (6/55) and 20.7% (6/29) of the eligible health center catchment areas.

### Inclusion criteria.

Any private provider who supplied antimalarial medicines and/or blood tests for malaria was eligible for inclusion in the survey. This included facilities with qualified health-care workers (e.g., nurses and pharmacists), some of whom also worked in public health facilities, as well as drug shops and grocery shops staffed by untrained sellers. Attempts were made to distinguish and select only “registered” or “trained” providers; however, this proved difficult due to the absence of up-to-date lists of such providers.

### Sample and data collection.

The study was conducted between November 2010 and January 2011. At each of the 12 health center catchment areas, the census survey teams visited the relevant local authorities to obtain lists of the names and location of known health facilities and outlets. The surveyors then tried to visit all the identified providers and in addition, visited any other providers who identified locally as being potential sources of antimalarials, including general stores and mobile providers.

The MC study was conducted during the same period as the overt census survey in all the selected health center catchment areas except one where dangerous roads made the district inaccessible in the interval between the census team and MC visits. Two nearby health center catchment areas were included in the MC study at the request of local authorities: Sala Krau in Pailin and Sotnikum in Siem Riep. In each health center catchment area, the MC study team attempted to visit all the legible outlets visited by the census study team. In one peri-urban area where there were a large number of providers and logistic constraints restricted the numbers that could be included in the MC study, 16 outlets were selected from the surveyor's list using a random number generator. The interval between the overt census survey and MC study was between 5 and 21 days.

The census survey was carried out by three teams of three or four surveyors and two supervisors. The MC study was carried out by a single team composed of two research assistants, three MC actors, and a supervisor. Surveyors and MC research assistants were given 1 week training including field-based practice. The three actors were local adult Khmer males who were given 3 days training to dress and act out scenarios as if they were forest workers, the main risk group for malaria in Cambodia.

In the census survey, surveyors obtained informed written consent from the most senior person in the outlets, and then used a structured questionnaire to collect information about the availability of antimalarials and mRDTs, the qualification of providers and their opinions and self-reported practice with regard to antimalarials and diagnosis. Observations were about the appearance of the outlet and also the medicine storage conditions. Based on surveyors' observations, outlets were categorized into different types based on size, types of goods and services provided, and presence of signs and certificates. Each outlet was georeferenced and samples of all ACAs that were offered were purchased, labeled, and sent for laboratory analysis.

During the MC study, the MCs presented themselves to each selected private provider either as patients themselves with symptoms of malaria or on behalf of a sick friend or relative. They were instructed to initially only give symptoms of malaria (fever, headache, body pain, chills) and to observe what the provider did and said, and to buy any medicines initially offered, before providing progressively more information eventually aimed specifically at trying to buy an artemisinin-based monotherapy. Immediately after the interaction, the MC was debriefed by a research assistant who audiotaped the debriefing and filled in a semistructured questionnaire.

All data collection tools were translated into Khmer and back translated to English, piloted and revised a number of times.

### Ethical approval and permissions.

Ethical approval for the study was granted from the National Ethics Committee for Health Research in Cambodia and the London School of Hygiene and Tropical Medicine Ethics Committee, United Kingdom (Ref: 5970). Results were reported to the Ministry of Health and relevant partners.

### Data entry and analysis.

Data were double entered in Microsoft Excel and checked for coding errors and consistency. Textual data were translated from Khmer into English and the two data sets were compared and inconsistencies if present resolved. Data analysis was conducted using STATA 11 (Stata Corp., College Station, TX) and Microsoft Excel.

Since there is no single accepted standard range for defining drugs as being poor quality in terms of %API, for the purposes of this paper, ACAs were defined as being poor quality if they contained < 85% or ≥ 115% of the stated API. This follows the recommendations by the U.S. Pharmacopeia Convention for the analysis of single-tablet samples. Identification of potential counterfeit medicines was based on packaging inspection and if chemical analysis identified either very low levels of API (< 20%) or the presence of other APIs. Analysis of degradation products was not performed on these samples, therefore, it was not possible to differentiate between medicines that were poor quality due to poor manufacturing practice versus degradation postmanufacture.

To identify predictors related to poor quality, χ^2^ tests were first performed using a univariate logistic regression model, with the effect of outlet and antimalarial associated characteristics on the outcome estimated using a Mantel–Haenszel test for comparing odds ratios (ORs). To increase statistical power and simplify interpretations, multilevel categorical variables were collapsed into binary ones. All variables with a *P* value ≤ 0.25 in the bivariate analysis were included in the multiple logistic regression model after they were checked for colinearity. Remaining nonsignificant predictors were introduced one at a time to detect for additional confounders. These remained in the model if the OR of other predictors in the model changed by greater than 20%. *P* values were adjusted for clustering at the district level and for stratification by containment zone using the STATA *svy* command.

### Sample and data handling.

All purchased medicines were labeled, stored, and transported under appropriate shipping conditions to Phnom Penh and then to the ACT Consortium Drug Quality project analytical laboratories in the United Kingdom and United States. Each sample was logged and labeled with a unique barcode linking it to a database containing detailed description of the packaging as well as details about the drugs.

### Packaging and laboratory analysis.

The packaging of each sample was scanned electronically and/or photographed. Analysis of the packaging was conducted by inspecting the package and comparing against authentic packaging wherever available.

Tablets were analyzed for the amount of API present using high-performance liquid chromatography (HPLC). HPLC analyses were conducted by pulverizing the tablets and extracting them in an appropriate solvent; artesunate and dihydroartemisinin were dissolved in methanol; mefloquine samples were dissolved in methanol/2.0 N hydrochloric acid (MeOH/2.0 N HCl; v/v) and piperaquine samples were dissolved in methanol/0.1 M HCl (1:1; v/v). Solvent extracts were sonicated followed by centrifuging, and the supernatant injected into the HPLC system for determining the amount of API present. Injectables (where the stated API carrier was coconut oil) were dissolved in methanol prior to HPLC.

HPLC using a Dionex Ultimate 3000 system (Thermofisher, Hemel Hempstead, United Kingdom) and separation was achieved using a GENESIS AQ 4 μm column (150 × 4.6 mm, Grace Materials Technologies, Cranforth, United Kingdom). The mobile phase was a gradient of ammonium formate (10 mM, pH 2.7) and acetonitrile (v/v; 60:40–85:15 over 7.0 minutes). A photodiode array unit (UV-PDA; DAD 3000, Thermofisher, Hemel Hempstead, UK) set at 204 nm for the artemisinin derivatives, 360 nm for piperaquine, and 259 nm for mefloquine was used as the detector. In all cases, the flow rate used was 1.0 mL/min. Calibration curves of each compound were generated by Dionex Chromeleon 7.2 chromatography data system software (Thermofisher, Hemel Hempstead, UK) using known amounts of the corresponding chemical standard (obtained from Sigma Aldrich, United Kingdom and Roche, Basel, Switzerland).

Samples were also sent to Georgia Institute of Technology, Atlanta, GA and the U.S. Centers for Disease Control and Prevention Laboratories, Atlanta, GA for HPLC confirmatory analysis and mass spectrometry screening, respectively.

Additional laboratory analysis details are available on request.

## Results

### General description.

**Figure 1.**
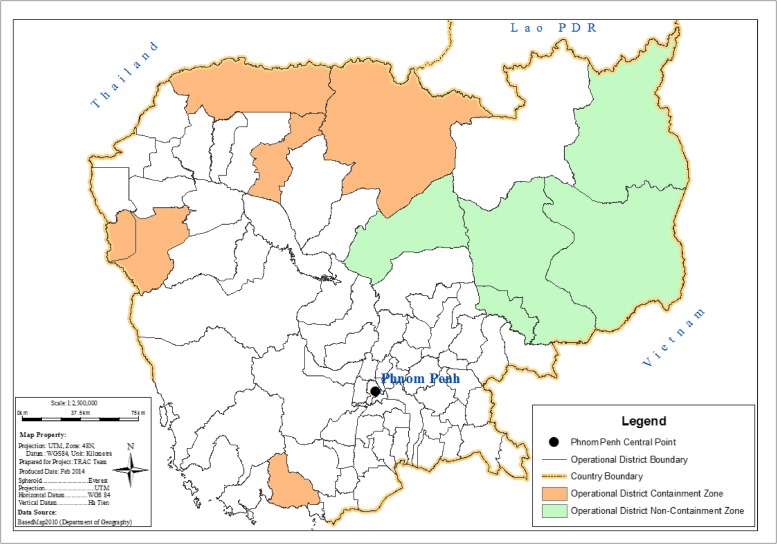
Map of the operational districts in which the study took place.

The randomized selection of health center catchment areas resulted in the six being within the containment zone strata all being in different provinces: Battambang, Pailin, Kampot, Oddar Meanchey, Preah Vihear, and Siem Riep. Outside of the containment zone, three of the six randomly selected health center catchment areas were in Kratie province and the other three were in Rattanakiri, Mondulkiri, and Kampong Thom provinces (Figure 1

).

In the census survey, a total of 430 outlets were screened. Two hundred and three outlets, reportedly sold antimalarial drugs of which 181 (89.2%) sold an ACA. The most common type of outlets was pharmacies (28.1%, *N* = 61) and grocery shops (25.3%, *N* = 55). In the MC study, a total of 211 interactions were conducted; these took place in 190 out of 203 (93.1%) outlets that were identified as selling antimalarial drugs during the census survey. A further 21 interactions were from the two health center catchment areas that were included in the MC study but not in the census. In the MC study, after the initial interaction, MCs were offered some medicines in three quarters of the cases (76.7%, 161/210), of which only 19.9% (32/161) were apparently for “malaria.” After the initial interaction, 86.3% (182/211) of MCs then gave more information to convince the provider to sell them an antimalarial, after which 45.6% (83/182) bought drugs that were apparently for malaria.^29^

### Description of the ACAs.

Overall 291 ACAs were bought and analyzed, 212 ACAs from the census survey and 79 from the MC survey (Table 1). The most common ACA was the co-blistered artesunate and mefloquine, especially Malarine, the co-blistered product, manufactured by Cipla in India and socially marketed by Population Services International.

**Figure 2.**
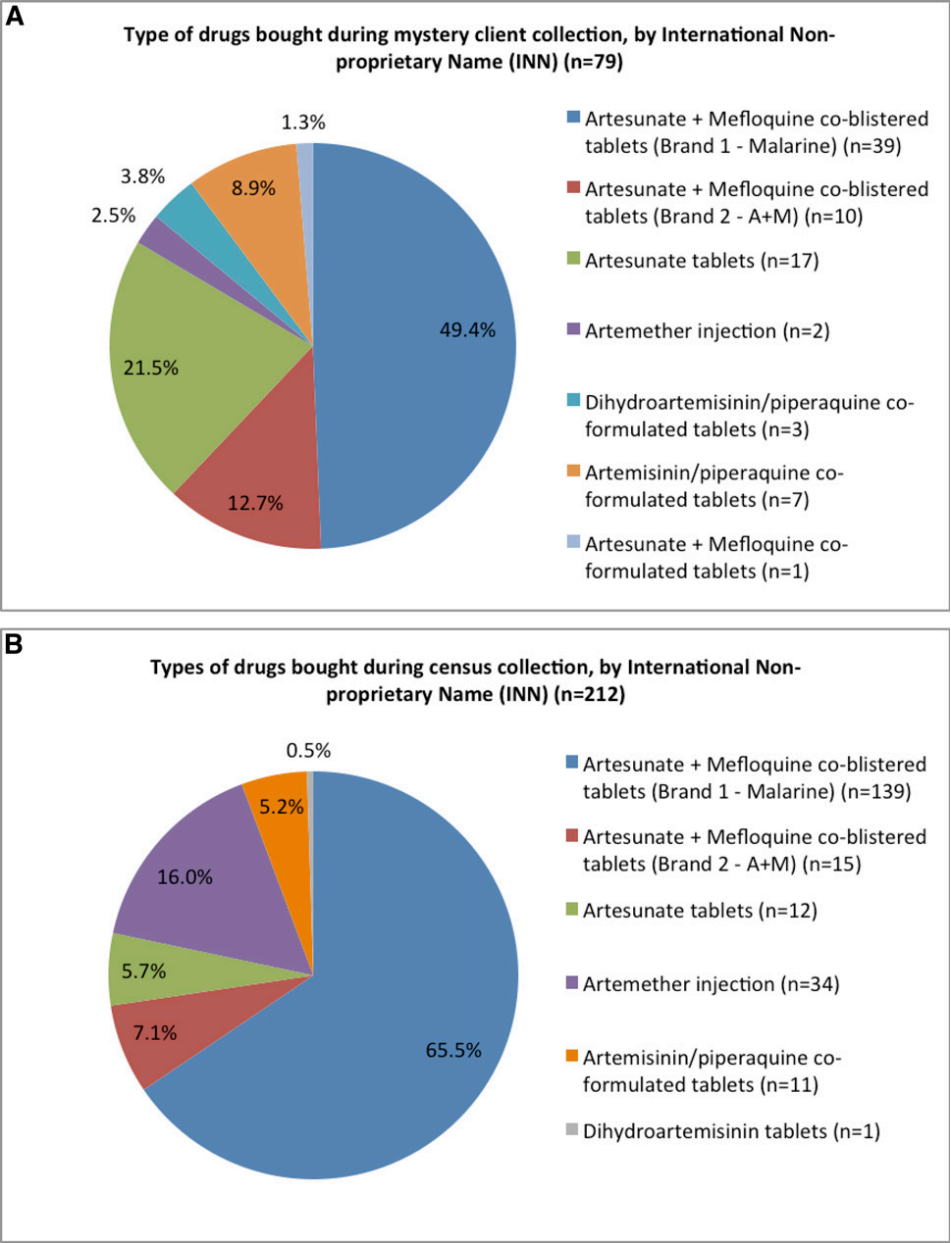
Pie charts to illustrate the types of artemisinin-containing antimalarials (ACAs) purchased through the census survey and mystery client (MC) study.

In the census survey, co-blistered artesunate and mefloquine accounted for three quarters (72.6%, 154/212) of ACAs, with the adult dose form of Malarine accounting for 54.7% (116/212) (Figure 2

). Injectable artemether, the first-line treatment of severe malaria, was the next most prevalent ACA (16.0%, 34/212), followed by oral artesunate monotherapy (5.7%, 12/212), and oral artemisinin co-formulated with piperaquine (5.2%, 11/212).

During MC visits, the MCs bought whatever medicines were offered, including packets containing a mix of drugs (“drug cocktails”) as well as whole packets of antimalarial medicines. A total of 190 cocktail packets were bought, of which 112 (58.9%) contained an antimalarial, 34 of which were ACAs. In addition, 45 ACAs were bought as complete blister packets, so that in all 79 ACAs were purchased. As in the census survey, the most common ACA form was as the adult dose form of Malarine (43.0%, *N* = 34/79). However, the next most common ACA was oral artesunate monotherapy (41.7%, *N* = 67/79), followed by oral artemisinin co-formulated with piperaquine (8.9%, *N* = 7), oral dihydroartemisinin co-formulated with piperaquine (3.8%, *N* = 3), and only two samples of injectable artemether.

There was a significantly lower likelihood of buying artesunate monotherapy through the overt surveyor approach compared with the MC (odds ratio [OR]: 0.2, 95% confidence interval [CI]: 0.09, 0.52, *P* = 0.0001). Conversely, there was a higher likelihood of buying injectable artemether through the overt survey, compared with the MC (OR: 7.45, 95% CI: 1.82, 65.26, *P* = 0.002).

Although there was a trend toward artesunate monotherapy being more likely to be sold in the non-containment area (*N* = 20) compared with the containment area (*N* = 8) (OR: 2.15, X^2^ *P* = 0.007), the small sample size limits the interpretation of these results.

### Expiry dates.

From the census survey, 9.9% (21/212) of drugs were found to be expired at the time of purchase with expiry dates ranging back to June 2009. No expiry date information was available for 3.3% (7/210) drugs. However, for drug bought by MCs, there was no expiry date information for 30.4% (24/79) drugs because they were sold outside of their original packaging. From the expiry date information that was available, 13.9% (11/79) drugs were expired at the time of purchase. Therefore, MC-purchased ACAs had twice the odds of being expired compared with those bought in the census study (OR: 2.19, 95% CI: 0.88, 5.16, *P*= 0.05). There was no association between stated brand name and whether a sample was expired.

### Packaging.

From the inspection and comparison of the packaging of the samples against available originals, there were no obvious falsified packages. There were a number of locally registered brands for which original packaging was not available to compare with; these included artesunate monotherapy tablets (Arquine^®^ and Artesunate from Bindinh pharma).

### API content analysis.

Two hundred and ninety one artemisinin derivatives were analyzed by HPLC. The most common form was artesunate tablets (80.1%, 233/291), most (87.1%, 203/233) of which were co-blistered with mefloquine with only one sample of co-formulated artesunate and mefloquine and the remainder 12.4% (29/233) of samples as the monotherapy. The second most common form was injectable artemether (12.4%, 36/291) followed by co-formulated tablets of artemisinin and piperaquine (6.2%, 18/291).

**Figure 3.**
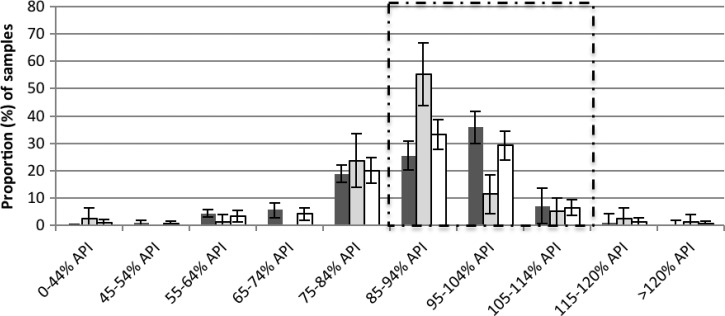
The percentage (mean and 95% confidence interval [CI]), of active pharmaceutical ingredient (API), as measured by high-performance liquid chromatography (HPLC), for all artemisinin-containing antimalarials (ACAs) by census collection (black) (*N* = 212), mystery client (MC) collection (grey) (*N* = 79), and overall (white) (*N* = 291). The proportion of samples that fell within the 85–115% API boundary is outlined by a dashed line.

All samples were found to contain the stated API (Figure 3

). Overall, 68.7% (*N* = 200/291) contained ≥ 85% and < 115% of the stated API and were considered of satisfactory quality for single tablet analysis, and 31.3% (*N* = 91) of samples were outside of this range and therefore considered poor quality (data not shown).

A quarter of medicines (72/291) were expired at the time of analysis, and of these, 40.3% (*N* = 29/72) were poor quality. For the drugs that were not expired at the time of analysis, 26.1% (49/188) were poor quality. Forty drugs expired between time of purchase and time of analysis but this did not affect the proportions for poor-quality drugs (Table 2).

For quality control purposes, 14 artesunate and 11 mefloquine samples were sent to the Centers for Disease Control and Prevention Laboratories, Atlanta, GA, for blinded interlab assay comparison using HPLC. The correlation of the results between both laboratories was high for both artesunate (*r* = 1.0) and mefloquine (*r* = 1.0) (data not shown). Overall, final results from both laboratories were consistently within 3–4% of each other and therefore considered unbiased.

#### By API type.

**Figure 4.**
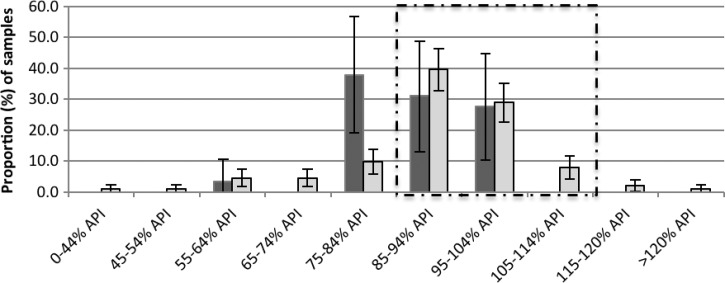
The percentage (mean and 95% confidence interval [CI]), of active pharmaceutical ingredient (API), as measured by high-performance liquid chromatography (HPLC), for artesunate tablets, sold alone as monotherapy (black) (*N* = 29), and co-blistered with mefloquine (grey) (*N* = 204). The proportion of samples that fell within the 85–115% API boundary is outlined by a dashed line.

**Figure 5.**
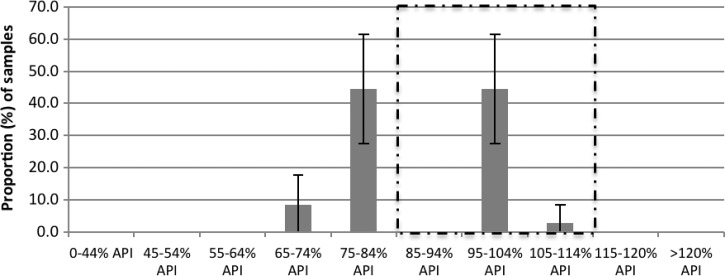
The percentage (mean and 95% confidence interval [CI]), of active pharmaceutical ingredient (API), as measured by high-performance liquid chromatography (HPLC), for injectable artemether samples (*N* = 36). The proportion of samples that fell within the 85–115% API boundary is outlined by a dashed line.

Three quarters 74.2% (173/233) of the oral artesunate tablets were of satisfactory quality (API ≥ 85% and < 115%). The tablets that were bought co-blistered with mefloquine had twice the odds (OR: 2.29, *P* = 0.04, 95% CI: 0.9, 5.5) of being of satisfactory quality compared with artesunate tablets that were bought on their own as a monotherapy (Figure 4

). For injectable artemether, of the 36 samples, only half (52.8%, 95% CI: 35.6, 69.9) of the samples were of satisfactory quality (Figure 5

).

There was considerable intra-batch and inter-batch variability in %API content. There was a trend toward drug quality being much more consistent in the first-line ACT (i.e., A + M and Malarine).

#### Partner drugs.

**Figure 6.**
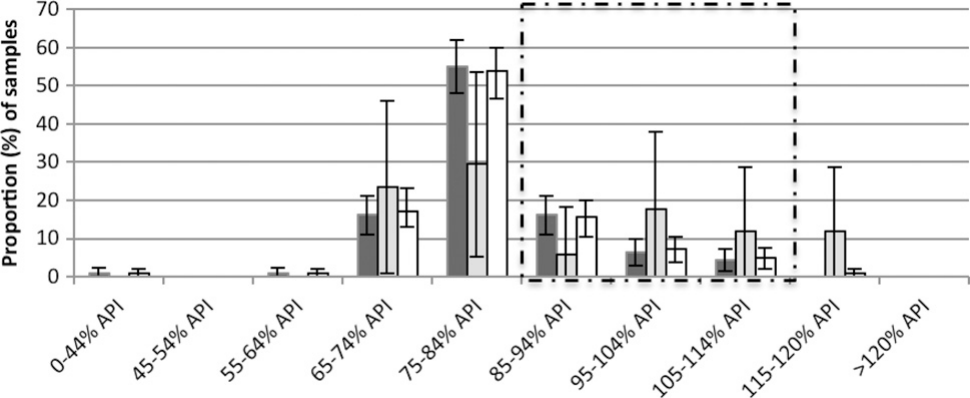
The percentage (mean and 95% confidence interval [CI]) of active pharmaceutical ingredient (API), as measured by high-performance liquid chromatography (HPLC), for mefloquine (black) (*N* = 204), piperaquine (grey) (*N* = 14), and total partner drug (white) (*N* = 218) samples. The proportion of samples that fell within the 85–115% API boundary is outlined by a dashed line.

A total of 225 partner drug samples were analyzed. Of the 203 mefloquine samples, only 54 (25.6%) were of satisfactory quality (API ≥ 85% and < 115%) so that for co-blistered-artesunate and mefloquine samples, when both drugs were taken into account, only 22.7% (*N* = 46/203) of samples contained the correct amount of API for both drugs (Table 3). For the piperaquine, six of the 17, (35.3%) samples were of satisfactory quality (Figure 6

).

#### Survey method.

Overall, MCs were not more likely than overt surveyors to collect poor-quality drugs (OR: 0.94, 95% CI: 0.51, 1.71, *P* = 0.84) except for artesunate monotherapy. The samples bought by a MC were more likely to be of poor quality than the few that were bought by a surveyor (OR 20.17 (95% CI: 1.84, 944.57, *P* = 0.002) (Supplemental Annex Tables 1 and 2).

### Risk factors for poor drug quality.

Table 4 shows the results of the bivariate and multivariate analysis for risk factors associated with poor-quality ACAs as defined by an API of < 85% or ≥ 115%.

By bivariate analysis, four variables were significantly associated with poor-quality ACAs: if the artemisinin derivative form was any other than oral artesunate (OR 3.21 [95% CI: 2.31, 4.48, *P* < 0.0001]); if the artesunate tablet was obtained as a monotherapy rather than co-blistered with mefloquine (OR 2.63 [95% CI: 1.34, 5.91, *P* = 0.010]); if the medicine was anything other than Malarine, (OR: 2.41, [95% CI: 1.26, 4.66, *P* = 0.012]); and if the medicine was in injectable form rather than tablet form (OR 2.63 [95% CI: 1.08, 6.42, *P* = 0.036]). There was no association between quality of samples collected from outlets within or outside of the containment area, qualification of providers, or between drugs that were stored inside a cabinet inside a shop compared with medicines stored elsewhere.

Following multivariate regression analysis, two variables were significant at *P* ≤ 0.05. Samples that were expired at the time of analysis had 2.56 (95% CI: 1.29, 5.07, *P* = 0.011) odds of being poor quality when compared with samples that were not expired and samples that cost ≤ 3,500 riels (US$0.85) had 1.65 odds (95% CI: 1.00, 2.72, *P* = 0.049) of being poor quality.

## Discussion

The Thai–Cambodia border has been the epicenter for antimalarial resistance for several decades and is now the focus of artemisinin resistance. Although there are many contributory factors, it is likely that the widespread prevalence in the past of artemisinin-based monotherapies and poor-quality antimalarials have played a significant part in the emergence of antimalarial drug resistance.

Recently, major efforts have been made to tackle the problem of poor-quality medicines through regulation, enforcement, education, and communication campaigns. However, there is a lack of detailed data documenting the impact of these interventions and in differentiating between prevalence of falsified medicines from other poor-quality medicines. This is of particular importance as combating these problems requires different strategies.^15^,^30^ This study attempts to address that gap. Furthermore, a randomized approach was used in this study, in contrast to studies in the past, which have adopted a convenience approach to sampling. Random sampling is recommended to obtain reliable estimates of prevalence^31^; to date this method has only been used in a few studies.^17^,^19^,^27^,^32^–^35^ Finally, this is the first study to compare two different methodologies for procuring medicines; overt survey versus a covert MC approach. Both methodologies have their strengths and weaknesses. Samples bought overtly by researchers may result in bias, due to shopkeepers holding back the drugs that are more likely to be falsified or poor quality. Purchasing drugs through MCs can avoid this potential bias but has other disadvantages, including limitations in terms of the number of different types of drugs that can be believably asked for.

### Key finding and implication for policy practice and research.

Fortunately, in this study no suspected falsified drugs were found and Malarine (the national first-line treatment of *Pf* malaria in Cambodia) was by far the most widely available antimalarial. This is extremely encouraging given the large number of falsified antimalarials previously reported.^4^,^21^ In addition, although some artesunate monotherapy was found, its prevalence was far less than prior to the ban on their sale. In addition, the quality of the artesunate tablets in the first-line co-blistered artesunate and mefloquine product was higher than in monotherapy products.

These findings are encouraging, suggesting a positive impact from some of the efforts made through the containment program. However, a number of other concerns are revealed. First, 31.3% of ACAs were considered poor quality at time of analysis, of which the majority (93.4%) contained too little rather than too much of the API. Around one tenth of medicines were past their expiry date at the time of purchase and drugs and samples that were expired had more than twice the odds of being poor quality.

The low levels of API found in poor-quality samples may be due to the degradation rather than problems with production. Medicines that were originally of good quality may degrade and become poor quality during routine transport and storage, especially if stored beyond their expiry date^36^ and if exposed to extremes of humidity and temperature.^12^,^37^,^38^ Artemisinin derivatives are inherently unstable and are very sensitive to heat and humidity. It is therefore essential to minimize the degradation process during transportation and storage to ensure that drugs remain useful for their active shelf life.^39^ There are little data on the quality of drugs past their expiry dates. However, in the absence of data, medicines used past their expiry date should be regarded as poor quality as they may be degraded.^28^ Future studies are needed to evaluate both the quality and the stability of drugs over time under routine storage conditions. However, in this current study, it is not possible to determine the cause of poor quality.

Although there has been much publicity about falsified medicines, the problem of poor-quality medicines has received less attention. However, it can be argued that they are as important for development of drug resistance and much more widespread.^30^,^40^ Falsified antimalarials often do not contain any of the stated active ingredients at all, although sometimes they can contain small quantities, possibly to evade detection. This can be potentially lethal to patients with malaria who take them in the belief that they are taking an effective antimalarial. However, falsified ACAs that do not contain any active ingredient do not exert selective artemisinin “drug pressure” on parasites and therefore do not contribute to the development of drug resistance. In contrast, poor-quality drugs and falsified drugs, which do contain sub-therapeutic amounts of the API, put the malaria patient in risk and also increase the risk of the selection of drug-resistant parasites.^12^,^15^,^16^,^37^,^41^

Since the completion of this study, Cambodia has switched its first-line treatment policy to the fixed dose combination of dihydroartemisinin and piperaquine for both uncomplicated *Pf* and *Pv* malaria. There were severe delays in the switch, resulting in the continued use of co-blistered artesunate and mefloquine and therefore the potential for patients to selectively take artesunate as monotherapy.^42^ Although the switch to a co-formulated ACT is welcome, there are some concerns about the stability of dihydroartemisinin,^43^ therefore, the quality of this product must be monitored closely.

A second finding that deserves discussion is the widespread availability of injectable artemether. Because it has been the recommended first-line treatment of severe malaria, it was not included in the ban on artemisinin-based monotherapies. Injections and infusions are very popular in Cambodia, as they are often perceived as being more powerful than oral preparations.^44^ It is not known whether the ban on oral artemisinins resulted in a shift to injectable preparations, but previous surveys have shown that it has been widely available since at least 2002.^45^–^48^ It could be argued that further research is required to document whether patients who are receiving injectable artemether are also receiving a full course of an ACT, and if not, what measures should be taken to ensure that they do. However, given the evidence of the superiority of intravenous artesunate^49^,^50^ and the recognition that complicated malaria should be treated in public health facilities, it would be more advisable to ensure referral and effective treatment of severe malaria to public health facilities and to discourage the use of parenteral artemisinins in the private sector except for pre-referral.

This study also confirmed some previously documented findings: the widespread availability of drug “cocktails” that often contain partial courses of antimicrobials, and an association between the cost of drugs and drug quality.^51^,^52^ Finally, although overall there was no significant difference in the quality of medicines bought by the two approaches, there were significant differences in the types of medicines bought. Less oral artesunate monotherapy was bought by the overt surveyors than MCs, perhaps reflecting providers' awareness of the ban on oral artemisinins and their reluctance to sell these overtly through fears of being reported or judged. Conversely, MCs obtained very few samples of injectable artemisinins as these are usually administered to the patient by providers and it was clearly ethically unacceptable to expect the MCs to subject themselves to the pain and risks associated with receiving unnecessary injections! It may be that different approaches are appropriate in different settings and further comparative studies are required to accurately describe the true prevalence of poor-quality medicines and establish standard methodological approaches to sample collection.

This study had a number of limitations. First, the sample size was relatively small. However, the selection was randomized and nationwide, and is therefore more robust and generalisable than most of the previously published studies on drug quality. Second, this study was only conducted in the private sector and not in public health facilities where the scale of the problem of expired and poor-quality drug remains unknown and deserves attention. Third, the definition of a threshold of poor quality drugs set at < 85% or ≥ 115% API may be criticized. Unfortunately, there is currently no accepted definition for poor-quality drugs that can be used to compare across different medicines. In the absence of an established threshold, we used a range that we believe is justifiable based on the USP guidelines, which allows for a wider range for analysis of single tablets. Finally, a cross-sectional study such as this only produces a snapshot in time and only reports on the quality of ACAs. Clearly, what is required is the strengthening of a routine surveillance system, which allows ongoing monitoring of all medicine quality. Much progress has been made in Cambodia toward this end with the support of many international partners including the WHO, Global Fund for AIDS, Tuberculosis, and Malaria, U. S. Pharmacopeia, the French embassy, USAID others. There is now strong multi-sectorial support including involvement of the Ministries of Interior, Police, Customs, and Education. However, the activities are subject to the stops and starts inevitably associated with short-term donor funding and other challenges including the lack of laboratory and human resources.

These problems are not unique to Cambodia. Ensuring that local drug regulatory agencies are strengthened and the medicine manufacturing process is improved can help prevent the problem of poor-quality antimalarials^53^; however, there is limited knowledge on the geography and trading patterns of poor quality medicines^41^,^54^ and, most importantly in most malaria-endemic countries, the capacity of most drug regulatory agencies is extremely limited allowing the manufacture and sale of poor-quality medicines without the risk of sanctions.^30^,^40^ There are insufficient testing facilities to analyze antimalarial drugs and poor consumer and health worker knowledge on drugs.^55^ The development and implementation of new analytical tools, which can be used in the field by drug inspectors and law enforcement officials are required to quickly assess whether medicines are of good quality.^56^ Although a number of alternatives are currently being explored, further work is required to evaluate their operational accuracy and feasibility.^57^–^60^ Whatever new tools are developed, there will always be a need for local capacity to implement them. Thirty percent of WHO member states have either no medicine regulation or a capacity that hardly functions^28^ and only 20% have fully operational regulatory mechanisms to test the quality specifications of medicines.^30^ Strengthening the capacity of national medicine regulatory authorities is a global health imperative.

## Supplementary Material

Supplemental Annex Tables.

## Figures and Tables

**Table 1:** Type of antimalarials analyzed by stated INN, stated brand name, stated manufacturer, and method of collection

Stated INN and formulation	Stated brand name (stated manufacturer)	MC collection *n* (%)	Census survey collection *n* (%)
Artesunate + Mefloquine co-blistered tablets	A + M	−	15
	(Unknown)	6 (7.6)	14 (6.6)
	(Cipla for PSI)	3 (3.8)	1 (0.5)
	(Roll Back Malaria)	1 (1.3)	0 (0.0)
	Malarine	−	−
	(Unknown)	35 (44.3)	135 (63.7)
	(Cipla for PSI)	4 (5.1)	4 (1.9)
Artesunate + Mefloquine co-formulated tablets	A + M	−	−
	(Farmanghuinos)	1 (1.3)	0 (0.0)
Artesunate tablets	Arquine 50	−	−
	(MS)	3 (3.8)	2 (0.9)
	Artesunat	−	−
	(Cong Ty Phan Duqc Pham Djch Vu Y Tekhanh nqi)	0 (0.0)	1 (0.5)
	(Unknown)	3 (3.8)	1 (0.5)
	Artesunate	−	−
	(Bindinh Pharma)	7 (8.9)	4 (1.9)
	(Canapharm)	1 (1.3)	1 (0.5)
	(Unknown)	3 (3.8)	3 (1.4)
Artemether injection	Artemedine	−	−
	(Kunming Pharmaceutical Corp)	0 (0.0)	1 (0.5)
	Artemether	−	−
	(Shanghai Pharmaceutical Industrial Corps)	0 (0.0)	18 (8.5)
	(Rotexmedica GmBH distributed by Dafra Pharmaceutical Corp)	1 (1.3)	0 (0.0)
	Artesiane 80	−	−
	(Unknown)	0 (0.0)	1 (0.5)
	(Rotexmedica GmBH distributed by Dafra Pharmaceutical Corp)	1 (1.3)	14 (6.6)
Artemisinin/piperaquine co-formulated tablets	Artequick	−	−
	(Artepharm Co. Ltd)	0 (0.0)	8 (3.8)
	(Unknown)	7 (8.9)	3 (1.4)
Dihydroartemisinin/piperaquine coformulated tablets	Duo-cotecxin	−	−
	(Zhejian Holley Nanhu)	3 (3.8)	0 (0.0)
Dihydroartemisinin tablets	Cotecxin	−	−
	(Beijing Holley-Cotec)	0 (0.0)	1 (0.5)
	Total	79 (100)	212 (100)

INN = international non-proprietary name; MC = mystery client; MS = medical supply.

**Table 2:** Expiry status and quality of samples by collection method, at time of purchase and time of analysis

		*n* (%)		
		All	Census survey collection	MC collection
	Total	291	212	79
	Of which poor quality	91 (31.3)	67 (31.6)	37 (30.4)
Status at time of purchase	Expired	32	21	11
	Of which poor quality	13 (40.6)	10 (47.6)	3 (27.3)
	Not expired	228	184	44
	Of which poor quality	65 (28.5)	55 (29.9)	10 (22.7)
	Don't know	31	7	24
	Of which poor quality	13 (41.9)	2 (28.6)	11 (25.8)
Status at time of analysis	Expired	72	53	19
	Of which poor quality	29 (40.3)	24 (45.3)	5 (26.3)
	Not expired	188	152	36
	Of which poor quality	49 (26.1)	41 (27.0)	8 (22.2)
	Don't know	31	7	24
	Of which poor quality	13 (41.9)	2 (28.6)	11 (45.8)

MC = mystery client.

**Table 3:**
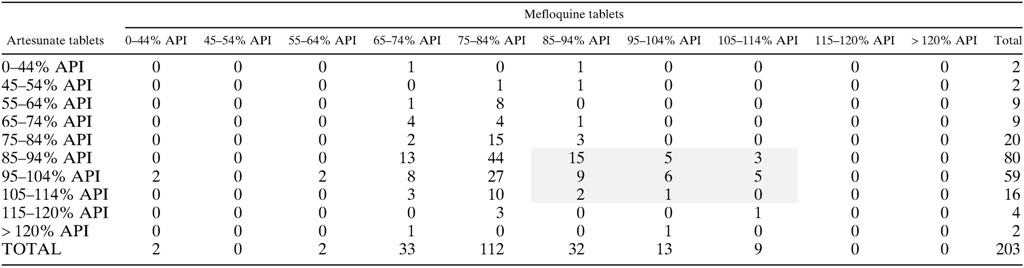
API content of co-blistered artesunate and mefloquine tablets as measured by HPLC*

API = actual pharmaceutical ingredient; HPLC = high-performance liquid chromatography.

* The shaded area denotes samples where both of the components are considered good quality with an API ≥ 85% or < 115%.

**Table 4:** Bivariate (crude) and multivariate (adjusted) model of association between poor quality ACAs (as defined by API < 85% or ≥ 115%) and exposure variables

	Total number of samples	Number (%) of poor quality samples (API < 85% or ≥ 115%)		Crude OR (95% CI)		*P* value	Adjusted OR (95% CI)		*P* value
	*N* = 291	*N* = 91 (%)							
Collection method									
Census	212	67	(31.6)	1.0	−	−	−	−	−
MC	79	24	(30.4)	0.96	(0.44, 2.08)	0.912	0.74	(0.31, 1.77)	0.471
Brand name									
Malarine (all age groups)	178	44	(24.7)	1.0	−	−	−	−	−
Other brands	113	47	(41.6)	2.41	(1.26, 4.66)	0.012**	1.65	(0.69, 3.98)	0.236*
Dose form									
Oral tablet	255	72	(28.2)	1.0	−	−	−	−	−
Liquid injection/ampoule	36	19	(52.8)	2.63	(1.08, 6.42)	0.036**	3.39	(0.94, 12.25)	0.061*
API									
Artesunate	233	60	(25.8)	1.0	−	−	−	−	−
Other APIs	58	31	(53.5)	3.21	(2.31, 4.48)	< 0.0001**	0.90	(0.22, 3.64)	0.877
Artesunate form†									
Co-blister with Mefloquine	204	48	(23.5)	1.0	−	−	−	−	−
Artesunate monotherapy	29	12	(41.4)	2.81	(1.34, 5.91)	0.010**	−	−	−
Location of outlet									
Inside containment area	134	41	(30.6)	1.0	−	−	−	−	−
Outside containment area	157	50	(31.9)	1.09	(0.52, 2.26)	0.802	1.09	(0.63, 1.87)	0.740
Expired at time of analysis?									
No	188	49	(26.1)	1.0	−	−	−	−	−
Yes	72	29	(40.3)	1.84	(0.92, 3.66)	0.080*	2.56	(1.29, 5.07)	0.011**
Price (Riel)									
> 3,500	202	62	(30.7)	1.0	−	−	−	−	−
≤ 3,500	77	27	(35.1)	1.16	(0.72, 1.88)	0.518	1.65	(1.00, 2.72)	0.049**
Qualification of outlet owner‡									
No training	35	13	(37.1)	1.0	−	−	−	−	−
At least some training	100	30	(30.0)	0.73	(0.37, 1.41)	0.305	−	−	−
Storage conditions‡									
Cabinet inside shop	192	60	(31.3)	1.0	−	−	−	−	−
Other storage conditions	16	7	(43.8)	1.71	(0.81, 3.60)	0.138*	−	−	−
Type of outlet‡									
Pharmacy	66	21	(31.8)	1.0	−	−	−	−	−
Other outlet types	142	46	(32.4)	1.03	(0.56, 1.87)	0.924	−	−	−

API = active pharmaceutical ingredient; ACAs = artemisinin-containing antimalarials; CI = confidence interval; OR = odds ratio.

* *P* ≤ 0.25.

** *P* < 0.05.

† Data collected for artesunate samples only.

‡ Data collected during Census only.

## References

[1] Wongsrichanalai C, Meshnick SR (2008). Declining artesunate-mefloquine efficacy against falciparum malaria on the Cambodia–Thailand border. Emerg Infect Dis.

[2] Yeung S, Patouillard E, Allen H, Socheat D (2011). Socially marketed rapid diagnostic tests and ACT in the private sector: ten years of experience in Cambodia. Malar J.

[3] Yanagisawa S, Mey V, Wakai S (2004). Comparison of health-seeking behaviour between poor and better-off people after health sector reform in Cambodia. Public Health.

[4] Rozendaal J (2000). Fake antimalarials circulated in Cambodia. Mekong Malaria Forum.

[5] Cambodia National Malaria Control Programme Annual Progress Reports 2009–2014.

[6] Littrell M, Gatakaa H, Phok S, Allen H, Yeung S, Chuor CM, Dysoley L, Socheat D, Spiers A, White C, Shewchuk T, Chavasse D, O'Connell KA (2011). Case management of malaria fever in Cambodia: results from national anti-malarial outlet and household surveys. Malar J.

[7] Noedl H, Socheat D, Satimai W (2009). Artemisinin-resistant malaria in Asia. N Engl J Med.

[8] Dondorp AM, Nosten F, Yi P, Das D, Phyo AP, Tarning J, Lwin KM, Ariey F, Hanpithakpong W, Lee SJ, Ringwald P, Silamut K, Imwong M, Chotivanich K, Lim P, Herdman T, An SS, Yeung S, Singhasivanon P, Day NP, Lindegardh N, Socheat D, White NJ (2009). Artemisinin resistance in *Plasmodium falciparum* malaria. N Engl J Med.

[9] Miotto O, Almagro-Garcia J, Manske M, Macinnis B, Campino S, Rockett KA, Amaratunga C, Lim P, Suon S, Sreng S, Anderson JM, Duong S, Nguon C, Chuor CM, Saunders D, Se Y, Lon C, Fukuda MM, Amenga-Etego L, Hodgson AV, Asoala V, Imwong M, Takala-Harrison S, Nosten F, Su XZ, Ringwald P, Ariey F, Dolecek C, Hien TT, Boni MF, Thai CQ, Amambua-Ngwa A, Conway DJ, Djimde AA, Doumbo OK, Zongo I, Ouedraogo JB, Alcock D, Drury E, Auburn S, Koch O, Sanders M, Hubbart C, Maslen G, Ruano-Rubio V, Jyothi D, Miles A, O'Brien J, Gamble C, Oyola SO, Rayner JC, Newbold CI, Berriman M, Spencer CC, McVean G, Day NP, White NJ, Bethell D, Dondorp AM, Plowe CV, Fairhurst RM, Kwiatkowski DP (2013). Multiple populations of artemisinin-resistant *Plasmodium falciparum* in Cambodia. Nat Genet.

[10] Degardin K, Roggo Y, Margot P (2014). Understanding and fighting the medicine counterfeit market. J Pharm Biomed Anal.

[11] Newton PN, Green MD, Fernandez FM, Day NP, White NJ (2006). Counterfeit anti-infective drugs. Lancet Infect Dis.

[12] Affum AO, Lowor S, Osae SD, Dickson A, Gyan BA, Tulasi D (2013). A pilot study on quality of artesunate and amodiaquine tablets used in the fishing community of Tema, Ghana. Malar J.

[13] Pincock S (2003). WHO tries to tackle problem of counterfeit medicines in Asia. BMJ.

[14] Cockburn R, Newton PN, Agyarko EK, Akunyili D, White NJ (2005). The global threat of counterfeit drugs: why industry and governments must communicate the dangers. PLoS Med.

[15] Newton PN, Green MD, Fernandez FM (2010). Impact of poor-quality medicines in the ‘developing’ world. Trends Pharmacol Sci.

[16] Hall KA, Newton PN, Green MD, De Veij M, Vandenabeele P, Pizzanelli D, Mayxay M, Dondorp A, Fernandez FM (2006). Characterization of counterfeit artesunate antimalarial tablets from southeast Asia. Am J Trop Med Hyg.

[17] Onwujekwe O, Kaur H, Dike N, Shu E, Uzochukwu B, Hanson K, Okoye V, Okonkwo P (2009). Quality of anti-malarial drugs provided by public and private healthcare providers in south-east Nigeria. Malar J.

[18] Newton PN, McGready R, Fernandez F, Green MD, Sunjio M, Bruneton C, Phanouvong S, Millet P, Whitty CJ, Talisuna AO, Proux S, Christophel EM, Malenga G, Singhasivanon P, Bojang K, Kaur H, Palmer K, Day NP, Greenwood BM, Nosten F, White NJ (2006). Manslaughter by fake artesunate in Asia–will Africa be next?. PLoS Med.

[19] Sengaloundeth S, Green MD, Fernandez FM, Manolin O, Phommavong K, Insixiengmay V, Hampton CY, Nyadong L, Mildenhall DC, Hostetler D, Khounsaknalath L, Vongsack L, Phompida S, Vanisaveth V, Syhakhang L, Newton PN (2009). A stratified random survey of the proportion of poor quality oral artesunate sold at medicine outlets in the Lao PDR: implications for therapeutic failure and drug resistance. Malar J.

[20] Dondorp AM, Newton PN, Mayxay M, Van Damme W, Smithuis FM, Yeung S, Petit A, Lynam AJ, Johnson A, Hien TT, McGready R, Farrar JJ, Looareesuwan S, Day NP, Green MD, White NJ (2004). Fake antimalarials in southeast Asia are a major impediment to malaria control: multinational cross-sectional survey on the prevalence of fake antimalarials. Trop Med Int Health.

[21] Lon CT, Tsuyuoka R, Phanouvong S, Nivanna N, Socheat D, Sokhan C, Blum N, Christophel EM, Smine A (2006). Counterfeit and substandard antimalarial drugs in Cambodia. Trans R Soc Trop Med Hyg.

[22] Newton P, Proux S, Green M, Smithuis F, Rozendaal J, Prakongpan S, Chotivanich K, Mayxay M, Looareesuwan S, Farrar J, Nosten F, White NJ (2001). Fake artesunate in southeast Asia. Lancet.

[23] Newton PN, Fernandez FM, Plancon A, Mildenhall DC, Green MD, Ziyong L, Christophel EM, Phanouvong S, Howells S, McIntosh E, Laurin P, Blum N, Hampton CY, Faure K, Nyadong L, Soong CW, Santoso B, Zhiguang W, Newton J, Palmer K (2008). A collaborative epidemiological investigation into the criminal fake artesunate trade in southeast Asia. PLoS Med.

[24] Aldhous P (2005). Counterfeit pharmaceuticals: murder by medicine. Nature.

[25] WHO (2010). World Malaria Report 2010.

[26] WHO (2011). Global Plan for Artemisinin Resistance Containment (GPARC).

[27] Odeniyi MA, Adegoke OA, Adereti RB, Odeku OA, Itiola OA (2003). Comparative analysis of eight brands of sulfadoxinepyrimethamine tablets. Trop J Pharm Res.

[28] Newton PN, Lee SJ, Goodman C, Fernandez FM, Yeung S, Phanouvong S, Kaur H, Amin AA, Whitty CJ, Kokwaro GO, Lindegardh N, Lukulay P, White LJ, Day NP, Green MD, White NJ (2009). Guidelines for field surveys of the quality of medicines: a proposal. PLoS Med.

[29] Yeung S, Chandler, C, Taberno, P, de Souza, M, Rada, O, Ngoun, C (2011). Good use of anti-malarials and rapid diagnostic tests in Cambodia (Guard) study report.

[30] Ravinetto RM, Boelaert M, Jacobs J, Pouget C, Luyckx C (2012). Poor-quality medical products: time to address substandards, not only counterfeits. Trop Med Int Health.

[31] Phanouvong SBN, Smine A (2004). Guidelines for Sampling of Antimalarial Drug Samples in the USP DQI Antimalarial Drug Quality Monitoring Project in Mekong Sub-Region Countries.

[32] Phanouvong S, Raymond C, Krech L, Dijiba Y, Mam B, Lukulay P, Socheat D, Sovannarith T, Sokhan C (2013). The quality of antimalarial medicines in western Cambodia: a case study along the Thai-Cambodian border. Southeast Asian J Trop Med Public Health.

[33] Taylor RB, Shakoor O, Behrens RH, Everard M, Low AS, Wangboonskul J, Reid RG, Kolawole JA (2001). Pharmacopoeial quality of drugs supplied by Nigerian pharmacies. Lancet.

[34] Kaur H, Goodman C, Thompson E, Thompson KA, Masanja I, Kachur SP, Abdulla S (2008). A nationwide survey of the quality of antimalarials in retail outlets in Tanzania. PLoS ONE.

[35] Ogwal-Okeng JW, Okello DO, Odyek O (1998). Quality of oral and parenteral chloroquine in Kampala. East Afr Med J.

[36] Pribluda VS, Barojas A, Anez A, Lopez CG, Figueroa R, Herrera R, Nakao G, Nogueira FH, Pianetti GA, Povoa MM, Viana GM, Gomes MS, Escobar JP, Sierra OL, Norena SP, Veloz R, Bravo MS, Aldas MR, Hindssemple A, Collins M, Ceron N, Krishnalall K, Adhin M, Bretas G, Hernandez N, Mendoza M, Smine A, Chibwe K, Lukulay P, Evans L (2012). Implementation of basic quality control tests for malaria medicines in Amazon Basin countries: results for the 2005–2010 period. Malar J.

[37] Newton PN, Green MD, Mildenhall DC, Plancon A, Nettey H, Nyadong L, Hostetler DM, Swamidoss I, Harris GA, Powell K, Timmermans AE, Amin AA, Opuni SK, Barbereau S, Faurant C, Soong RC, Faure K, Thevanayagam J, Fernandes P, Kaur H, Angus B, Stepniewska K, Guerin PJ, Fernandez FM (2011). Poor quality vital anti-malarials in Africa: an urgent neglected public health priority. Malar J.

[38] Keoluangkhot V, Green MD, Nyadong L, Fernandez FM, Mayxay M, Newton PN (2008). Impaired clinical response in a patient with uncomplicated falciparum malaria who received poor-quality and underdosed intramuscular artemether. Am J Trop Med Hyg.

[39] Nogueira FH, Moreira-Campos LM, Santos RL, Pianetti GA (2011). Quality of essential drugs in tropical countries: evaluation of antimalarial drugs in the Brazilian Health System. Rev Soc Bras Med Trop.

[40] Caudron JM, Ford N, Henkens M, Mace C, Kiddle-Monroe R, Pinel J (2008). Substandard medicines in resource-poor settings: a problem that can no longer be ignored. Trop Med Int Health.

[41] Tabernero P, Newton PN (2012). The WWARN antimalarial quality surveyor. Pathog Glob Health.

[42] Yeung S, Van Damme W, Socheat D, White NJ, Mills A (2008). Access to artemisinin combination therapy for malaria in remote areas of Cambodia. Malar J.

[43] Haynes RK, Chan HW, Lung CM, Ng NC, Wong HN, Shek LY, Williams ID, Cartwright A, Gomes MF (2007). Artesunate and dihydroartemisinin (DHA): unusual decomposition products formed under mild conditions and comments on the fitness of DHA as an antimalarial drug. ChemMedChem.

[44] Tawfik L (2005). Mosquitoes, Malaria and Malarine: A Qualitative Study on Malaria Drug Use in Cambodia.

[45] Khol V, Mao B, Saphonn V, An U, Bruce J, Meek S, Lines J, Cox J (2005). Report of the Cambodia National Malaria Baseline Survey, 2004.

[46] Dysoley L, Rithea L, Bunkea T, Babu S, Sim K, Nguon C, Sochea D, Thompson M, Bruce J, de Beyl CZ, Cox J, Sintasath D, Meek S (2010). Cambodia Malaria Survey Report.

[47] An SU, Mao B, Saphonn V, Bruce J, Meek S, Lines J, Cox J (2007). Cambodia Malaria Survey Report.

[48] PSI (2011). Kingdom of Cambodia Outlet Survey Report.

[49] Dondorp AM, Fanello CI, Hendriksen IC, Gomes E, Seni A, Chhaganlal KD, Bojang K, Olaosebikan R, Anunobi N, Maitland K, Kivaya E, Agbenyega T, Nguah SB, Evans J, Gesase S, Kahabuka C, Mtove G, Nadjm B, Deen J, Mwanga-Amumpaire J, Nansumba M, Karema C, Umulisa N, Uwimana A, Mokuolu OA, Adedoyin OT, Johnson WB, Tshefu AK, Onyamboko MA, Sakulthaew T, Ngum WP, Silamut K, Stepniewska K, Woodrow CJ, Bethell D, Wills B, Oneko M, Peto TE, von Seidlein L, Day NP, White NJ (2010). Artesunate versus quinine in the treatment of severe falciparum malaria in African children (AQUAMAT): an open-label, randomised trial. Lancet.

[50] Dondorp A, Nosten F, Stepniewska K, Day N, White N (2005). Artesunate versus quinine for treatment of severe falciparum malaria: a randomised trial. Lancet.

[51] Bate R, Jin GZ, Mathur A (2011). Does price reveal poor-quality drugs? Evidence from 17 countries. J Health Econ.

[52] Bate R, Jin GZ, Mathur A (2012). Counterfeit or Substandard? Assessing Price and Non-Price Signals of Drug Quality.

[53] El-Duah M, Ofori-Kwakye K (2012). Substandard artemisinin-based antimalarial medicines in licensed retail pharmaceutical outlets in Ghana. J Vector Borne Dis.

[54] Talisuna AO, Karema C, Ogutu B, Juma E, Logedi J, Nyandigisi A, Mulenga M, Mbacham WF, Roper C, Guerin PJ, D'Alessandro U, Snow RW (2012). Mitigating the threat of artemisinin resistance in Africa: improvement of drug-resistance surveillance and response systems. Lancet Infect Dis.

[55] Nayyar GM, Breman JG, Newton PN, Herrington J (2012). Poor-quality antimalarial drugs in southeast Asia and sub-Saharan Africa. Lancet Infect Dis.

[56] Fernandez FM, Hostetler D, Powell K, Kaur H, Green MD, Mildenhall DC, Newton PN (2011). Poor quality drugs: grand challenges in high throughput detection, countrywide sampling, and forensics in developing countries. Analyst (Lond).

[57] Ioset JR, Kaur H (2009). Simple field assays to check quality of current artemisinin-based antimalarial combination formulations. PLoS ONE.

[58] Deisingh AK (2005). Pharmaceutical counterfeiting. Analyst (Lond).

[59] Bate R, Hess K (2010). Anti-malarial drug quality in Lagos and Accra—a comparison of various quality assessments. Malar J.

[60] Bate R, Tren R, Mooney L, Hess K, Mitra B, Debroy B, Attaran A (2009). Pilot study of essential drug quality in two major cities in India. PLoS ONE.

