# Supplementary material for: Quality of Antimalarials at the Epicenter of Antimalarial Drug Resistance: Results from an Overt and Mystery Client Survey in Cambodia
Source: Am J Trop Med Hyg. 2015 Jun 3;92(Suppl 6):39–50. doi: 10.4269/ajtmh.14-0391 (PMC4455075; doi:10.4269/ajtmh.14-0391)
Supplement: Supplementary file 1 [file SD3.pdf]

Supplemental Annex Table 1

Percentage API of ACAs collected by census survey

			0–44% API	45–54% API	55–64% API	65–74% API	75–84% API	85–94% API	95–104% API	105–114% API	115–120% API	> 120% API				
Stated brand name	Stated API and formulation	*n*	*n* (%)	*n* (%)	*n* (%)	*n* (%)	*n* (%)	*n* (%)	*n* (%)	*n* (%)	*n* (%)	*n* (%)	Mean	SD	Minimum	Maximum
Artemedine	Artemether liquid injection (80 mg/mL)	1	0 (0)	0 (0)	0 (0)	1 (100)	0 (0)	0 (0)	0 (0)	0 (0)	0 (0)	0 (0)	74.1	–	–	–
Artemether	Artemether liquid injection (80 mg/mL)	18	0 (0)	0 (0)	0 (0)	2 (11.1)	16 (88.9)	0 (0)	0 (0)	0 (0)	0 (0)	0 (0)	79.2	3.47	69.6	83.6
Artesiane 80	Artemether liquid injection (80 mg/mL)	15	0 (0)	0 (0)	0 (0)	0 (0)	0 (0)	0 (0)	15 (100)	0 (0)	0 (0)	0 (0)	98	1.76	95.7	101.4
Cotecxin	Dihydroartemisinin tablets (60 mg)	1	1 (100)	0 (0)	0 (0)	0 (0)	0 (0)	0 (0)	0 (0)	0 (0)	0 (0)	0 (0)	33.2	–	–	–
Artequick	Co-blistered artemisinin 62.5 mg and piperaquine 375 mg	11	0 (0)	0 (0)	0 (0)	0 (0)	6 (54.5)	4 (36.4)	1 (9.1)	0 (0)	0 (0)	0 (0)	86.7	5.19	80.2	97.5
Arquine 50	Artesunate tablets (50 mg)	2	0 (0)	0 (0)	0 (0)	0 (0)	1 (50.0)	0 (0)	1 (50.0)	0 (0)	0 (0)	0 (0)	90.1	7.82	84.6	95.7
A + M1	Co-blistered artesunate 50 mg and mefloquine 250 mg	1	0 (0)	0 (0)	0 (0)	0 (0)	0 (0)	1 (100)	0 (0)	0 (0)	0 (0)	0 (0)	92.3	–	–	–
A+ M2	Co-blistered artesunate 50 mg and mefloquine 250 mg	3	0 (0)	0 (0)	0 (0)	0 (0)	0 (0)	2 (66.7)	1 (33.3)	0 (0)	0 (0)	0 (0)	93.8	1.5	92.4	95.4
A + M3	Co-blistered artesunate 50 mg and mefloquine 250 mg	1	0 (0)	0 (0)	0 (0)	0 (0)	0 (0)	1 (100)	0 (0)	0 (0)	0 (0)	0 (0)	94.6	–	–	–
A + M4	Co-blistered artesunate 50 mg and mefloquine 250 mg	4	0 (0)	0 (0)	0 (0)	0 (0)	0 (0)	3 (75.0)	1 (25.0)	0 (0)	0 (0)	0 (0)	94.7	1.73	93.5	97.2
A + M5	Co-blistered artesunate 50 mg and mefloquine 250 mg	6	0 (0)	0 (0)	0 (0)	0 (0)	0 (0)	6 (100)	0 (0)	0 (0)	0 (0)	0 (0)	88.3	2.72	85.8	93.6
Malarine adolescent	Co-blistered artesunate 50 mg and mefloquine 250 mg	11	0 (0)	0 (0)	0 (0)	2 (18.2)	8 (72.7)	1 (9.1)	0 (0)	0 (0)	0 (0)	0 (0)	77.7	4.53	69.2	87.9
Malarine adult	Co-blistered artesunate 50 mg and mefloquine 250 mg	116	0 (0)	2 (1.7)	9 (7.8)	6 (5.2)	8 (6.9)	31 (26.7)	45 (38.8)	12 (10.3)	2 (1.7)	1 (0.9)	92.1	14.62	50.3	122.9
Malarine child	Co-blistered artesunate 50 mg and mefloquine 250 mg	8	0 (0)	0 (0)	0 (0)	0 (0)	0 (0)	1 (12.5)	5 (62.5)	2 (25.0)	0 (0)	0 (0)	95.9	2.6	92.2	98
Malarine	Co-blistered artesunate 50 mg and mefloquine 250 mg	4	0 (0)	0 (0)	0 (0)	1 (25.0)	1 (2.5)	1 (25.0)	0 (0)	1 (25.0)	0 (0)	0 (0)	88.8	15.18	73.8	108.8
AS (Bindinh Pharma)	Artesunate tablets (50 mg)	4	0 (0)	0 (0)	0 (0)	0 (0)	0 (0)	1 (25.0)	3 (75.0)	0 (0)	0 (0)	0 (0)	95.9	2.6	92.2	98
AS (Canada Pharm)	Artesunate tablets (50 mg)	1	0 (0)	0 (0)	0 (0)	0 (0)	0 (0)	1 (100)	0 (0)	0 (0)	0 (0)	0 (0)	93.2	–	–	–
A + M (unknown)	Co-blistered artesunate 50 mg and mefloquine 250 mg	0	0 (0)	0 (0)	0 (0)	0 (0)	0 (0)	0 (0)	0 (0)	0 (0)	0 (0)	0 (0)	–	–	–	–
AS (unknown)	Artesunate tablets (50 mg)	3	0 (0)	0 (0)	0 (0)	0 (0)	0 (0)	1 (33.3)	2 (66.7)	0 (0)	0 (0)	0 (0)	97.4	3.55	93.9	101
ArtesunAT	Artesunate tablets (50 mg)	2	0 (0)	0 (0)	0 (0)	0 (0)	0 (0)	0 (0)	2 (100)	0 (0)	0 (0)	0 (0)	101.3	1.6	100.2	102.4

API = active pharmaceutical ingredient; ACAs = artemisinin-containing antimalarials; SD = standard deviation.

Supplemental Annex Table 2

Percentage API of ACAs collected by MCs

			0–44% API	45–54% API	55–64% API	65–74% API	75–84% API	85–94% API	95–104% API	105–114% API	115–120% API	> 120% API				
Stated brand name	Stated API and formulation	*n*	*n* (%)	*n* (%)	*n* (%)	*n* (%)	*n* (%)	*n* (%)	*n* (%)	*n* (%)	*n* (%)	*n* (%)	Mean	SD	Minimum	Maximum
Artemether	Artemether liquid injection (80 mg/mL)	1	0 (0)	0 (0)	0 (0)	0 (0)	0 (0)	0 (0)	0 (0)	1 (100)	0 (0)	0 (0)	105.2	–	–	–
Artesiane 80	Artemether liquid injection (80 mg/mL)	1	0 (0)	0 (0)	0 (0)	0 (0)	0 (0)	0 (0)	1 (100)	0 (0)	0 (0)	0 (0)	103.9	–	–	–
Duo-cotecxin	Dihydroartemisinin tablets (60 mg)	3	0 (0)	0 (0)	0 (0)	0 (0)	0 (0)	0 (0)	1 (33.3)	2 (66.7)	0 (0)	0 (0)	104.7	4.16	100.0	108.0
Artequick	Co-blistered artemisinin 62.5 mg and piperaquine 375 mg	7	0 (0)	0 (0)	0 (0)	0 (0)	5 (71.4)	2 (28.6)	0 (0)	0 (0)	0 (0)	0 (0)	83.2	3.96	78.5	90.4
Arquine 50	Artesunate tablets (50 mg)	3	0 (0)	0 (0)	1 (33.3)	0 (0)	2 (66.7)	0 (0)	0 (0)	0 (0)	0 (0)	0 (0)	72.8	8.9	62.5	78.2
A + M5	Co-blistered artesunate 50 mg and mefloquine 250 mg	3	0 (0)	0 (0)	0 (0)	0 (0)	2 (66.7)	1 (33.3)	0 (0)	0 (0)	0 (0)	0 (0)	83.8	1.55	82.2	85.3
Malarine adolescent	Co-blistered artesunate 50 mg and mefloquine 250 mg	1	0 (0)	0 (0)	0 (0)	0 (0)	0 (0)	0 (0)	1 (100)	0 (0)	0 (0)	0 (0)	96.3	–	–	–
Malarine adult	Co-blistered artesunate 50 mg and mefloquine 250 mg	34	1 (2.9)	0 (0)	0 (0)	0 (0)	0 (0)	25 (73.5)	4 (11.8)	1 (2.9)	2 (5.9)	1 (2.9)	93.4	12.71	40.6	122
Malarine	Co-blistered artesunate 50 mg and mefloquine 250 mg	4	0 (0)	0 (0)	0 (0)	0 (0)	0 (0)	2 (50.0)	2 (50.0)	0 (0)	0 (0)	0 (0)	94.7	0.57	94.1	95.3
AS (Bindinh Pharma)	Artesunate tablets (50 mg)	7	0 (0)	0 (0)	0 (0)	0 (0)	5 (71.4)	2 (28.6)	0 (0)	0 (0)	0 (0)	0 (0)	81.3	3.45	77.8	86.4
AS (Canada Pharm)	Artesunate tablets (50 mg)	1	0 (0)	0 (0)	0 (0)	0 (0)	0 (0)	1 (100)	0 (0)	0 (0)	0 (0)	0 (0)	85.9	–	–	–
A + M (unknown)	Co-blistered artesunate 50 mg and mefloquine 250 mg	8	1 (12.5)	0 (0)	0 (0)	0 (0)	1 (12.5)	6 (75.0)	0 (0)	0 (0)	0 (0)	0 (0)	82.1	16.3	43.2	93.6
AS (unknown)	Artesunate tablets (50 mg)	3	0 (0)	0 (0)	0 (0)	0 (0)	1 (33.3)	2 (66.7)	0 (0)	0 (0)	0 (0)	0 (0)	86.2	4.41	82.5	91.1
ArtesunAT	Artesunate tablets (50 mg)	3	0 (0)	0 (0)	0 (0)	0 (0)	2 (66.7)	1 (33.3)	0 (0)	0 (0)	0 (0)	0 (0)	86	4.92	82.9	91.7

API = active pharmaceutical ingredient; ACAs = artemisinin-containing antimalarials; MCs = mystery clients; SD = standard deviation.
